# Analysis of various factors affecting pupil size in patients with glaucoma

**DOI:** 10.1186/s12886-017-0564-6

**Published:** 2017-09-16

**Authors:** Ji Woong Park, Bong Hui Kang, Ji Won Kwon, Kyong Jin Cho

**Affiliations:** 1 Department of ophthalmology, College of Medicine, Dankook University, 119, Dandae-ro, Dnognam-gu, Cheonan-si, Chungnam 31119 Republic of Korea; 2 Department of Neurology, College of Medicine, Dankook University, Cheonan, Republic of Korea; 3 Department of ophthalmology, Myongji Hospital, Seonam University College of Medicine, 697-24 Hwajung-dong, Deokyang-gu, Goyang-si, Kyeonggi-do 14075 Republic of Korea

**Keywords:** Glaucoma, Humphrey static perimetry, Pupil size

## Abstract

**Background:**

Pupil size is an important factor in predicting post-operative satisfaction. We assessed the correlation between pupil size, measured by Humphrey static perimetry, and various affecting factors in patients with glaucoma.

**Methods:**

In total, 825 eyes of 415 patients were evaluated retrospectively. Pupil size was measured with Humphrey static perimetry. Comparisons of pupil size according to the presence of glaucoma were evaluated, as were correlations between pupil size and various factors, including age, logMAR best corrected visual acuity (BCVA), retinal nerve fiber layer (RNFL) thickness, spherical equivalent, intraocular pressure, axial length, central corneal thickness, white-to-white, and the kappa angle.

**Results:**

Pupil size was significantly smaller in glaucoma patients than in glaucoma suspects (*p* < 0.001) or the normal group (*p* < 0.001). Pupil size decreased significantly as age (*p* < 0.001) and central cornea thickness (*p* = 0.007) increased, and increased significantly as logMAR BCVA (*p* = 0.02) became worse and spherical equivalent (*p* = 0.007) and RNFL thickness (*p* = 0.042) increased. In patients older than 50 years, pupil size was significantly larger in eyes with a history of cataract surgery.

**Conclusions:**

Humphrey static perimetry can be useful in measuring pupil size. Pupil size was significantly smaller in eyes with glaucoma. Other factors affecting pupil size can be used in a preoperative evaluation when considering cataract surgery or laser refractive surgery.

## Background

The pupil regulates the amount of light reaching the retina and minimizes chromatic aberration and spherical aberration to maximize visual perception [1]. Pupil size is an important factor in predicting post-operative satisfaction. In particular, it is important to accurately measure pupil size before performing cataract surgery or laser-related corneal refractive surgery, because pupil size can be an important cause of side effects, such as night light glaring and monocular double phase, which can occur after surgery [2].

The pupil is affected by the state of the sphincter muscle, according to the autonomic nerves distributed in the iris, and may vary from person to person depending on age and gender, even in the same lighting environment. Generally, pupil size changes with aging and it has been reported to decrease continuously until the 60s [3]. Pupil size decreases significantly after cataract surgery versus before surgery, and it is significantly smaller than in those without diabetes mellitus before and after cataract surgery [4, 5]. In the case of glaucoma, pupil size varies according to the type of intraocular pressure (IOP) lowering eyedrops [6–8]. Pupil size increases significantly from hyperopic astigmatism to myopic astigmatism, and a negative correlation with spherical aberration has been reported [9]. Additionally, the ‘pupil fluctuation phenomenon’ means that pupil size can change even in a given light environment. Thus, various factors must be considered when measuring pupil size [1].

Pupil size increases when the illumination environment changes from bright to dark. Clinically, it is important to understand pupil changes exactly in dark versus bright illuminance. Especially, when the pupil is exposed to dark illumination, the pupil becomes larger than the optic size of the intraocular lens, resulting in glaring and halo. This kind of side effect is the cause of much discomfort among patients [10].

Various devices using optical amplification, infrared rays, photographs, and video can be used to measure pupil size. For example, a ‘pupil measurement table’ can be used to measure pupil size relatively easily without high costs. The Colvard automatic pupilometer (Oasis Medical, Glendora, CA, USA) is comparatively cheap, portable, and relatively easy to use. However, because examiners must read their own eyes, this technique involves error depending on the examiner and the disadvantage of having a learning curve.

Using the principle of a Scheimpflug camera with placido-disc topographer coupled with ORBScan II (Bausch & Lomb, Orbtek Inc., Salt Lake City, UT, USA), Sirius can automatically measure pupil size when the patient looks at the central point of view inside the machine. ORBScan II can measure pupil size in bright illumination (photopic vision) and Sirius can measure pupil size under various illumination conditions, including bright (photopic), weak bright (mesopic), and dark (scotopic) illumination. In addition to these measuring instruments, a field of vision test (30–2 Swedish Interactive Thresholding Algorithm (SITA) standard) by Humphrey static perimetry (Carl Zeiss Meditec, Dublin, CA) can measure pupil size in terms of the amount of light reflected in the pupil when the patient is looking at the center of the machine. This is done in a controlled environment that is close to mesopic illumination.

In this study, we measured pupil size in weak bright illumination, using Humphrey static perimetry, to investigate various factors affecting pupil size and compared pupil size in normal, glaucoma suspect, and glaucoma groups.

## Methods

### Subjects and protocols

Patients who visited Dankook University Hospital from 2011 to 2015 and underwent measurements of mean pupil size using Humphrey static perimetry and mean RNFL thickness at the same time were selected and a retrospective study was performed. Patients with ophthalmological, medical, or medication-related illnesses that may affect pupil size, ocular disease, or trauma history (except glaucoma or refractive error) and an ocular surgery history (other than cataract or refractive surgery) were excluded. Both eyes were included in this study. In total, 825 eyes of 415 patients were selected. The values of logarithm of the minimum angle of resolution (logMAR), best corrected visual acuity (BCVA), spherical aberration, IOP, axial length, central corneal thickness, white-to-white, and kappa angle were included for each patient.

All patients were classified into one of three groups: normal, glaucoma suspect, and glaucoma. If there was no family history of glaucoma and no other retinal or optic nerve disease, we classified the subject as normal. If there was no visual field impairment in Humphrey static perimetry, IOP > 22 mmHg or the optic disc ratio was >0.5, or the difference in optic disc ratio between the eyes was ≥0.2, we classified the subject as a glaucoma suspect. If there was visual field impairment by Humphrey static perimetry and glaucomatous optic disc changes, we classified the subject into the glaucoma group.

### Biometrics

**Fig. 1:**
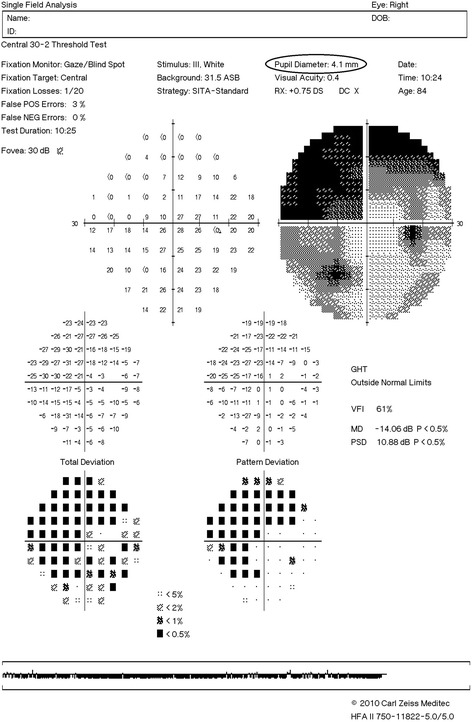
The visual field examination of right eye in glaucoma patient. The diameter of the right eye pupil is 4.1 mm

Humphrey static perimetry measures pupil size through the amount of light reflected to the pupil in a controlled environment close to mesopic illumination, with an average of 54 lx, maintained in a constant darkroom environment. The minimum measurement unit is 0.1 mm. In our hospital, all patients were instructed to look at the viewpoint in the machine in a controlled darkroom environment when undergoing a visual field examination (Fig. 1).

The mean RNFL thickness of the patients at the time of the perimetry measurements was measured with the Optic disc cube 200 × 200 scan mode of a spectral-domain optical coherence tomography (Cirrus HD-OCT, ver. 5; Carl Zeiss Meditec, Dublin, USA). Visual acuity was measured using a ‘Hahn Chun Suk’ visual acuity chart and converted to logMAR visual acuity. The IOP was typically measured using a Goldmann applanation tonometer; when the patient was not cooperative, a non-contact tonometer (tonometer CT-80, TOPCON, Japan) or iCare rebound tonometry (Tiolat Oy, Finland) was used.

Axial length was measured using an IOL Master (Zeiss, Oberkochen, Germany). Non-contact specular microscopy (SP-3000P, Topcon) was used to measure central corneal thickness, and corneal topography was performed using an ORBScan II (Bausch & Lomb, Orbtek Inc., Salt Lake City, UT, USA).

### Statistical analysis

SPSS software (ver. 18.0; SPSS Inc., Chicago, IL) was used to analyze the data. The independent *t*-test was used for comparisons of the normal, glaucoma suspect, and glaucoma groups. A simple linear regression test was used for the correlation analysis of various factors affecting pupil size in all patients. The mean pupil size was calculated and we compared each value based on the upper and lower median values. We compared pupil size according to the presence of refractive surgery in patients under 30 years and cataract surgery in patients over 50. We assessed whether these results were consistent with previous studies. A *p*-value <0.05 was considered to indicate statistical significance.

## Results

The mean age of the 415 subjects (825 eyes) was 45.57 ± 17.33 years and the mean logMAR BCVA was 0.06 ± 0.15. The mean pupil size was 5.52 ± 1.15 mm, the mean RNFL thickness was 81.58 ± 15.15 μm, and the mean spherical aberration was −1.74 ± 2.76 (Table 1).

**Table 1:** Patient demographics and characteristics including pupil size

	Number (eyes)	Mean ± SD	Min	Max
Age (years)	825	45.57 ± 17.33	9	85
LogMAR BCVA	825	0.06 ± 0.15	0	2
Pupil size (mm)	825	5.52 ± 1.15	2.2	8.8
Mean RNFL thickness (μm)	825	81.58 ± 15.15	36	127
Spherical equivalent	825	−1.74 ± 2.76	−12.88	7.37
IOP (mmHg)	825	13.78 ± 3.56	2	32
Axial length (mm)	240	24.79 ± 1.51	20.79	28.87
CCT (μm)	495	517.14 ± 41.85	386	663
WTW (mm)	112	11.58 ± 1.07	10.9	13.3
A kappa	112	4.25 ± 1.66	1.25	9.7

*BCVA* best corrected visual acuity, *RNFL* retinal nerve fiber layer, *IOP* intraocular pressure, *CCT* central corneal thickness, *WTW* white-to-white, *Min* minimum value, *Max* maximum value

All patients were classified according to the presence or absence of glaucoma. In total, 157 eyes (19.03%) were normal, 330 eyes (40%) were glaucoma suspects, and 338 eyes (40.97%) had glaucoma. The mean ages of these groups were 32.09, 43.73, and 53.63 years, respectively. Thus, age tended to increase with glaucoma. The mean IOPs were 13.61 ± 2.90, 14.09 ± 3.43, and 13.56 ± 3.93 mmHg, respectively, which were not significantly different. The average pupil sizes were 5.80 ± 1.10, 5.61 ± 1.10, and 5.28 ± 1.18 mm, respectively. Mean pupil size tended to decrease significantly from normal to glaucoma. The mean RNFL thicknesses were 91.80 ± 10.19, 87.95 ± 11.08, and 70.61 ± 13.56 μm, respectively; it tended to decrease from normal to glaucoma (Table 2).

**Table 2:** Mean pupil size and other parameters in each group

Subgroup	Number (eyes)	Age (years)	IOP (mmHg)	Pupil size (mm)	Mean RNFL thickness (μm)	Spherical equivalent
Total	825	45.57 ± 17.33	13.78 ± 3.56	5.52 ± 1.15	81.58 ± 15.15	−1.74 ± 2.76
Normal	157	32.09 ± 11.83	13.61 ± 2.90	5.80 ± 1.10	91.80 ± 10.19	−2.97 ± 3.01
Glaucoma suspect	330	43.73 ± 16.43	14.09 ± 3.43	5.61 ± 1.10	87.95 ± 11.08	−1.49 ± 2.49
Glaucoma	338	53.63 ± 15.91	13.56 ± 3.93	5.28 ± 1.18	70.61 ± 13.56	−1.41 ± 2.74

Values are means ± SD; IOP: intraocular pressure; RNFL: retinal nerve fiber layer

**Fig. 2:**
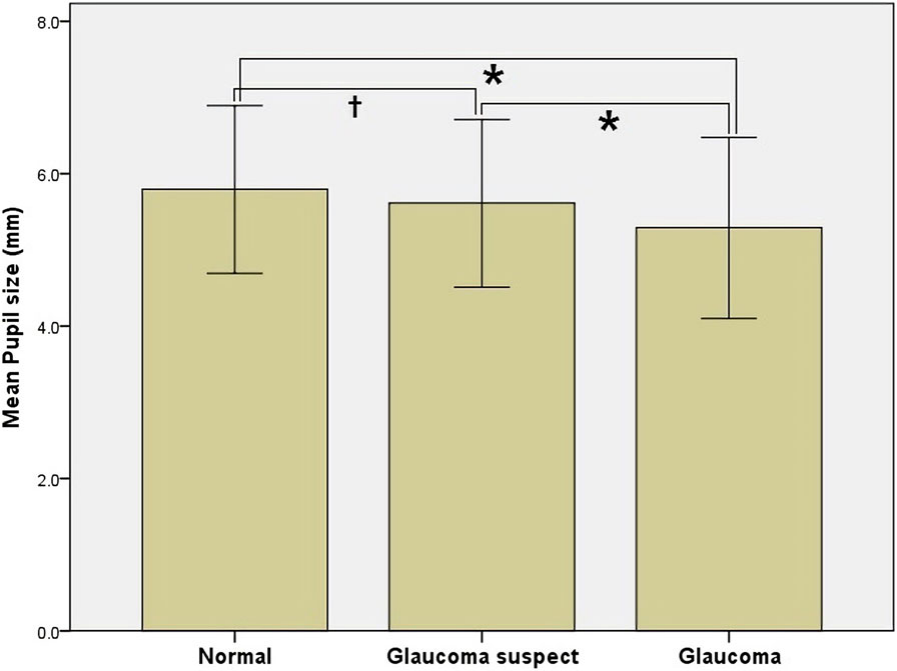
Comparison of average pupil size between three groups. The mean pupil size in the glaucoma group is statistically significantly smaller than in normal and glaucoma suspect groups. *p-value < 0.001. † *p-value >* 0.05

When comparing the mean pupil size in the two of the three groups according to the presence or absence of glaucoma, there was no statistically significant difference in glaucoma suspects versus normal controls (*p* = 0.087). However, the mean pupil size in the glaucoma group was statistically significantly smaller than in normal and glaucoma suspect groups (*p* < 0.001 and <0.001, respectively; Fig. 2).

In regression analysis comparing the relationship between various measured factors and pupil size, mean pupil size decreased significantly with age (*y* = −0.010 *x* + 5.962, where *x* = age (years) and *y* = mean pupil size (mm); *p* < 0.001), and it decreased as the logMAR BCVA decreased (*y* = −0.615 *x* + 5.550. *x* = logMAR BCVA, *p* = 0.02). Pupil size also decreased with increasing central corneal thickness (*y* = −0.003 *x* + 7.079, *x* = central corneal thickness (μm), *p* = 0.02). Pupil size was correlated positively with spherical aberration (*y* = 0.039 *x* + 5.584. *x* = spherical equivalent, *p* = 0.007), and weakly, but also positively, with mean RNFL thickness (*y* = 0.005 *x* + 5.076, *x* = retinal nerve fiber layer, *p* = 0.042). Pupil size did not have a significant relationship with other factors assessed including IOP, axial length, white-to-white, or kappa angle values (*p* = 0.326, 0.157, 0.872, and 0.129, respectively; Table 3).

**Table 3:** Correlation between mean pupil size and each factor

	Number	*y* = a *x* + b^a^	*p*-value^b^	Beta^c^
Age (year)	825	*y* = −0.010 *x* + 5.962	<0.001	−0.147
IOP (mmHg)	825	*y* = −0.011 *x* + 5.668	0.326	−0.034
Log MAR BCVA	825	*y* = −0.615 *x* + 5.550	0.02	−0.081
Spherical Equivalent	825	*y* = 0.039 *x* + 5.584	0.007	0.094
Axial length (mm)	240	*y* = 0.073 *x* + 3.753	0.157	0.092
CCT (um)	495	*y* = −0.003 *x* + 7.079	0.02	−0.104
WTW (mm)	112	*y* = −0.016 *x* + 6.080	0.872	−0.015
A kappa	112	*y* = 0.097 *x* + 5.483	0.129	0.144
RNFL thickness (um)	825	*y* = 0.005 *x* + 5.076	0.042	0.071

*BCVA* best corrected visual acuity, *CCT* central corneal thickness, *WTW* white-to-white, *RNFL* retinal nerve fiber layer

^a^ *y* = independent factor, *x* = mean pupil size

^b^simple linear regression test

^c^standardized coefficient beta by simple linear regression test

The median pupil size was 5.6 mm: all subjects were divided into two groups based on the median value: pupil size larger than 5.6 mm (‘large pupil’) and pupil size smaller than 5.6 mm (‘small pupil’). We explored possible differences in the values of the various factors between groups. The average pupil size of the small pupil group was 4.61 ± 0.73 mm and the average pupil size of the large pupil group was 6.50 ± 0.58 mm (*p* < 0.001). The mean age was significantly higher in the small pupil group than in the large pupil group, 47.75 ± 17.53 versus 43.19 ± 16.81 (p < 0.001), and the logMAR BCVA values were 0.07 ± 0.16 and 0.04 ± 0.15, respectively, which was significantly better in the large pupil group (*p* = 0.012). Mean RNFL thicknesses were 80.52 ± 14.89 μm and 82.73 ± 15.36 μm, respectively, which was significantly thicker in the large pupil group (*p* = 0.037). The mean spherical aberration values were −1.99 ± 2.91 and −1.46 ± 2.56, respectively, indicating that the small pupil group had a more myopic tendency (*p* = 0.005). The central corneal thicknesses were 521.14 ± 41.16 μm and 513.22 ± 42.23 μm, respectively, i.e., thicker in the small pupil group (*p* = 0.035). No other significant differences were observed between the groups in the other factors examined, including IOP, axial length, white-to-white value, and kappa value (Table 4).

**Table 4:** Comparison of mean pupil size and other factors between two groups divided by median pupil size (5.6 mm)

	Small pupil (≤ 5.6 mm)	Large pupil (> 5.6 mm)	*p*-value^a^
Number (eyes)	431	394	
Age (years)	47.75 ± 17.53	43.19 ± 16.81	<0.001
LogMAR BCVA	0.07 ± 0.16	0.04 ± 0.15	0.012
Pupil size (mm)	4.61 ± 0.73	6.50 ± 0.58	<0.001
Mean RNFL (μm)	80.52 ± 14.89	82.73 ± 15.36	0.037
Spherical equivalent	−1.99 ± 2.91	−1.46 ± 2.56	0.005
IOP^c^ (mmHg)	13.90 ± 3.49	13.63 ± 3.64	0.277
Axial length (mm)	24.83 ± 1.69 (*n* = 117)	24.75 ± 1.33 (*n* = 123)	0.7
CCT (μm)	521.14 ± 41.16 (*n* = 245)	513.22 ± 42.23 (*n* = 250)	0.035
WTW (mm)	11.61 ± 0.41 (*n* = 45)	11.56 ± 1.34 (*n* = 67)	0.807
A kappa	3.90 ± 1.65 (*n* = 45)	4.50 ± 1.64 (*n* = 67)	0.064

Values are means ± SD; *BCVA* best corrected visual acuity, *RNFL* retinal nerve fiber layer thickness, *IOP* intraocular pressure, *CCT* central corneal thickness, *WTW* white-to-white

^a^independent *t*-test

When 106 patients under 30 years old were assessed according to presence of previous refractive surgery, 12 (11.32%) patients underwent refractive surgery and their mean pupil size was 5.93 ± 1.12 mm. The other 94 (88.68%) patients had no history of previous refractory surgery; their mean pupil size was 5.87 ± 1.13 mm, and there was no significant difference between the groups (*p* = 0.856). When the 369 patients over 50 years old were divided according to presence of previous cataract surgery, 93 (25.20%) patients had undergone cataract surgery and their mean pupil size was 5.13 ± 1.27 mm. The other 276 (74.80%) patients had a history of cataract surgery; their mean pupil size was 5.54 ± 1.11 mm. Thus, the mean pupil size was significantly smaller in patients with previous cataract surgery (*p* = 0.004; Table 5).

**Table 5:** Comparison of mean pupil size between two groups according to history of refractive surgery

	Surgery history	Number (eyes)	Mean pupil size	*p*-value
Refractive surgery in patients under 30 years of age	yes	12	5.93 ± 1.12	0.856^a^
	no	94	5.87 ± 1.13	
Cataract surgery in patients over 50 years of age	yes	93	5.13 ± 1.27	0.004^b^
	no	276	5.54 ± 1.11	

^a^ normality was confirmed, so an independent *t*-test was used

^b^ independent *t*-test

## Discussion

This study investigated factors affecting pupil size, which is an important consideration in ophthalmic surgery. The pupil size was measured using Humphrey static perimetry. Various methods have been developed to measure pupil size: pupil measurement tables, the Colvard automatic pupilometer (Oasis Medical, Glendora, CA, USA), and Sirius (Costruzione Strumenti Oftalmici, Florence, Italy), which uses the principle of a Scheimpflug camera with placido-disc topographer coupled with ORBScan II (Bausch & Lomb), which are known to measure slightly differently [11].

Humphrey static perimetry measures the pupil size through the amount of light reflected to the pupil when the patient is looking at the internal gaze point in a controlled darkroom environment when performing visual field testing. The brightness of the light in mesopic illumination corresponds to 0.05 to 50 lx; Wachler and Krueger [12] reported a mean mesopic pupil size of 4.95 mm, and Chaglasian et al. [13] reported 5.17 mm.

In this study, the background illuminance when measuring pupil size with Humphrey static perimetry was about 54 lx, when measured at the center of the hemisphere using an illuminometer (DX-100, Takemura, Japan) [14]. This is near the level light of mesopic illumination, and the pupil size measured at this time may also be considered a mesopic pupil size. In fact, the mean pupil size measured in this way was 5.52 ± 1.15 mm, slightly larger than previous reports (Table 1). In a Korean study, Ko et al. [10]. reported that the mean pupil size measured under 5 lx illumination using a Colvard automatic pupilometer was >7 mm in adults under 60 years. It is also reasonable to consider the difference between the inherent measurement method of this machine and the illumination standard.

Pupil size may be associated with intraocular lens diameter and with night light glaring, blurring, decreased contrast sensitivity, and monocular diplopia, which are possible postoperative complications [1]. Especially in the field of refractive surgery, the correlation between pupil size and visual discomfort in a dark night environment after surgery is an important topic [15]. Generally, it is known that the larger pupil size in dark illumination, the more inconvenient night visual discomfort is after refractive surgery [16]. This suggests that the size of the scotopic or mesopic pupil size is more important than in the photopic state, clinically [1]. The present study is meaningful because it collected measurement values using the recent technique of Humphrey static perimetry, and took into consideration various factors related to pupil size in the clinically important mesopic condition.

Pupil size tended to decrease, from normal to glaucoma suspects, and glaucoma patients. This tendency can be considered in terms of the influence of glaucoma itself or of external factors. Glaucoma is a disease that causes progressive loss of retinal ganglion cells and optic nerve axons, which can lead to visual field defects. It is also known to reduce pupil reflexes compared with normal eyes. According to previous studies, in the presence of glaucoma, when the pupil contracts, there is lower amplitude, and slower velocity and acceleration [17–19]. Accordingly, when the opposite eye is normal or has a relatively less progressed glaucoma, relative afferent pupillary defect may be observed. The degree of this may be associated with the degree of visual field defect and optic nerve damage [20]. Mean RNFL thickness also decreased from normal to glaucoma in this study (Table 2) and the RNFL thickness was positively correlated with pupil size (Table 3). The effect of this on the pupil reflex can be considered to be related to the small pupil size in glaucoma patients.

In addition to glaucoma disease itself, pupil size may be affected by the type of IOP-lowering eye drops used. According to previous reports, pupil size decreased significantly when thymoxamine hydrochloride-based IOP-lowering eye drops were used [6], and brimonidine tartrate-based eye drops also reduced pupil size under mesopic illumination [7]. However, apraclonidine hydrochloride-based IOP-lowering agents caused pupil size to increase [8]. The present study was limited because it did not explore these effects. Additionally, given that the pupil size decreases continuously until age 60, the significant difference in age from normal to glaucoma may be a confounding factor.

With regard to relationships between pupil size and the various factors considered, pupil size decreased significantly with age, as reported in previous studies. Pupil size was also slightly positively correlated with mean RNFL thickness. A previous study found that the smaller the pupil size, the better the uncorrected vision; [7] thus, if the diameter of pupil becomes too large, the value of spherical aberration may increase and visual acuity may decrease. In contrast, in the present study, the smaller the pupil size, the worse logMAR BCVA (*y* = −0.615 *x* + 5.550. *x* = logMAR BCVA, *p* = 0.02). However, the beta value of the standardization coefficient, which actually indicates the influence, was as low as −0.081. Considering that other variables (other than age) were not corrected, this may not be a significant result. In previous studies, spherical aberration and pupil size were significantly negatively correlated [9]. However, a significant positive correlation was observed in the present study (*y* = 0.039 *x* + 5.584. *x* = spherical equivalent, *p* = 0.007). The correlation coefficient and the beta of the standardized coefficient were very small, 0.039 and 0.094, respectively, and other variables were not considered. The central corneal thickness and pupil size were also weakly, but significantly, positively correlated. Considering that prostaglandin has been reported to reduce central corneal thickness significantly [21], this may be due to an effect of IOP-lowering agents. There was a significant difference in the correlation between the two groups based on the median pupil size. The items that did not have a significant correlation before division also had no significant difference with this classification (Table 3).

There was no statistically significant difference in terms of a history of refractive surgery and pupil size in patients under 30 years old. Given that the number of specimens was too small and the postoperative follow-up period was shorter than that after cataract surgery, further systematic and ongoing research is needed. Pupil size is known to decrease significantly after cataract surgery versus before surgery [5]. Moreover, it has been reported that during the surgery, the sensitive dilator muscle of pupil is affected by the mechanical manipulation of the iris and pupil function is changed [22]. In fact, in this study, mean pupil size was significantly smaller in patients older than 50 years who had undergone cataract surgery (Table 5).

This study had several limitations. First, we did not verify the error and the utility of pupil size measured by Humphrey static perimetry in comparison with other pupil size measurement systems. Second, the number of specimens increased with both eyes involved, but correlation between two eyes in one patient was not corrected for. Third, the types and effects of IOP-lowering agents used in glaucoma suspects and glaucoma patients were not considered. Fourth, there was a lack of data, such as axial length, corneal horizontal diameter, kappa angle, and the number of patients undergoing refractive surgery younger than 30 years old. More research is needed to explore the various factors affecting pupil size.

## Conclusions

Humphrey static perimetry may be a new and useful measurement system for determining pupil size in a photopic or mesopic environment, which is an important consideration in correcting cataract and laser keratoplasty. Considering that the presence of glaucoma, logMAR BCVA, age, spherical aberration, and central corneal thickness are correlated significantly with pupil size, this may be a useful tool for predicting postoperative pupil size. More systematic research in the future will be helpful in predicting pupil size based on the various factors that affect pupil size.

## Abbreviations

BCVA: Best corrected visual acuity
IOP: Intraocular pressure
LogMAR: Logarithm of the minimum angle of resolution
RNFL: Retinal nerve fiber layer
SITA: Swedish interactive thresholding algorithm
